# Synergistic algae-bacteria interactions in a novel membrane aeration biofilm system: performance and microbial function

**DOI:** 10.3389/fmicb.2026.1900925

**Published:** 2026-07-29

**Authors:** Guoshuo Chen, Yixin Pan, Zijia Bai, Yanling Zheng, Yanjie Wei

**Affiliations:** 1 CCCC FHDI Engineering Co. Ltd, Guangzhou, China; 2 School of Energy and Environmental Engineering, Hebei University of Technology, Tianjin, China; 3 State Key Laboratory of Estuarine and Coastal Research, East China Normal University, Shanghai, China; 4 Tianjin Research Institute of Water Transport Engineering, Tianjin, China

**Keywords:** algae-bacteria symbiosis, extracellular polymeric substances, low C/N wastewater, membrane-aerated biofilm reactor, nitrogen removal

## Abstract

Low carbon-to-nitrogen (C/N) ratio wastewater poses a major challenge to biological nitrogen removal due to insufficient electron donors for denitrification. In this study, an algae-bacteria membrane-aerated biofilm reactor (AB-MABR) was established to enhance nitrogen removal under carbon-limited conditions, and its performance was compared with that of a conventional bacterial MABR (B-MABR). The results showed that the AB-MABR achieved superior pollutant removal performance, with COD, NH_4_^+^-N, and TN removal efficiencies being 4.0, 21.9, and 12.3% higher, respectively, than those of the B-MABR. Overall, AB-MABR outperformed B-MABR in pollutant removal. The removal efficiencies of COD, NH_4_^+^-N, and TN were 92.3, 77.2, and 66.6%, respectively, which were markedly higher than those achieved by B-MABR (88.8, 55.3, and 54.3%). The incorporation of microalgae significantly enhanced microbial metabolic activity, as evidenced by higher ATP content, electron transport system activity (ETSA), and cytochrome c (Cyt-c) levels. Meanwhile, EPS production increased by 25% in the AB-MABR, accompanied by greater accumulation of protein-like and humic-like substances. SEM and CLSM analyses revealed that microalgae promoted the formation of a denser and more stratified biofilm with higher biomass and stronger structural stability. Metagenomic analysis further demonstrated that pathways associated with microbial metabolism, secondary metabolite biosynthesis, and environmental adaptation were enriched in the AB-MABR system, indicating enhanced metabolic potential and ecological resilience. Overall, microalgal incorporation strengthened electron transfer, stimulated EPS secretion, improved biofilm development, and enhanced microbial metabolic functions, thereby promoting nitrogen transformation and removal under low C/N conditions. These findings provide new insights into the synergistic mechanisms of algae–bacteria biofilms and demonstrate the potential of AB-MABR technology for sustainable nitrogen removal from carbon-limited wastewater.

## 1 Introduction

The continuous increase in nitrogen loading from domestic and industrial sources has led to the widespread occurrence of low carbon-to-nitrogen (C/N) ratio wastewater, posing a significant challenge for biological nitrogen removal. Conventional nitrification–denitrification processes rely on sufficient organic carbon as electron donors for heterotrophic denitrification (Fu et al., 2022). However, under carbon-limited conditions, denitrification becomes kinetically constrained, resulting in incomplete nitrogen removal and reduced process efficiency (Sun et al., 2010). Although external carbon addition is commonly adopted to overcome this limitation, it inevitably increases operational costs and process complexity, underscoring the need for more sustainable nitrogen removal strategies.

The algae-bacteria symbiotic system has garnered significant attention due to its highly efficient nitrogen removal under low- or non-aerated conditions (Liang et al., 2025). Within this consortium, microalgae directly assimilate substantial nitrogen and fuel bacterial metabolism via photosynthetic oxygen evolution, thereby minimizing the need for mechanical aeration and external carbon dosing. Furthermore, microalgae secrete abundant extracellular polymeric substances (EPS); these carbon-rich polymers can serve as alternative electron donors to drive bacterial denitrification (Park et al., 2013). Thus, this symbiotic system is expected to be a promising technology for nitrogen removal from low carbon-to-nitrogen ratio wastewater. However, the stability of the algal-bacterial system remains a critical bottleneck. The small size and poor settleability of algal cells render the biomass susceptible to washout, whereas the nutritional mode between algae and heterotrophic bacteria often perturb microbial equilibrium (Bose et al., 2019). In addition, conventional aeration introduces excessive shear stress and oxygen fluctuations, which can disrupt biofilm formation and weaken microbial interactions, thereby limiting overall performance (Tang et al., 2016).

To overcome these challenges, membrane-aerated biofilm reactors (MABRs) present a promising alternative owing to their unique mass transfer characteristics. Oxygen delivery through gas-permeable membranes completely mitigates conventional aeration-induced turbulence, shielding the fragile algal-bacterial matrix from excessive shear stress (Bongartz et al., 2023). Concurrently, the resulting counter-diffusion of oxygen and bulk substrates generates distinct, coexisting redox gradients within the biofilm (Li et al., 2023). This spatial segregation successfully harmonizes the mismatched growth kinetics and nutritional modes of photoautotrophs and heterotrophs, sustaining microbial equilibrium. Additionally, the membrane surface serves as an ideal substratum for robust biofilm development, effectively counteracting the washout of small-sized algal cells. Consequently, coupling the algal-bacterial consortium with an MABR system is expected to mitigate algal biomass washout, alleviate competitive exclusion by heterotrophic bacteria, and shield algae from aeration-induced shear stress. The integration of algae into MABRs is poised to fundamentally alter biofilm architecture and nitrogen transformation pathways, particularly under low C/N ratios. Nevertheless, the mechanisms linking biofilm stratification, electron transfer processes, and nitrogen conversion pathways in algae-bacteria MABR systems remain poorly understood. In particular, how microalgae-induced changes in biofilm architecture regulate electron allocation and subsequently affect nitrification–denitrification coupling under low C/N conditions has rarely been investigated. Therefore, elucidating these interactions is essential for optimizing nitrogen removal performance and advancing the practical application of AB-MABR technology.

In this study, an AB-MABR was developed to investigate nitrogen removal under carbon-limited conditions. The objectives were to: (i) evaluate the removal performance of COD, NH_4_^+^-N, and TN; (ii) investigate the effects of microalgal incorporation on microbial activity, electron transfer, and EPS production; and (iii) elucidate the mechanisms underlying enhanced nitrogen removal through integrated analyses of biofilm structure and microbial metabolic functions.

## 2 Materials and methods

### 2.1 The cultivation of algae and bacteria

The microalga used in this study was obtained from a commercial supplier and cultivated in BG-11 medium under laboratory conditions. Based on microscopic morphological observations, the culture was identified as Chlorella sp. As the culture was purchased from a commercial source, no accession number or culture collection information was available. The light intensity was maintained at 5000 lx, and the temperature was set at 26 °C. The culture was cultivated under an 18 h light/6 h dark photoperiod for 10 days, which increased the mixed liquor suspended solids (MLSS) concentration to approximately 1,500 mg/L.

The activated sludge used in this study was obtained from a municipal wastewater treatment plant operated by Shandong Qixin Environmental Engineering Co., Ltd. Prior to the experiments, the sludge was cultivated and enriched with synthetic wastewater in a sequencing batch reactor (SBR) mode for 15 days.

### 2.2 Experiment set-up and operation

**Figure 1:**
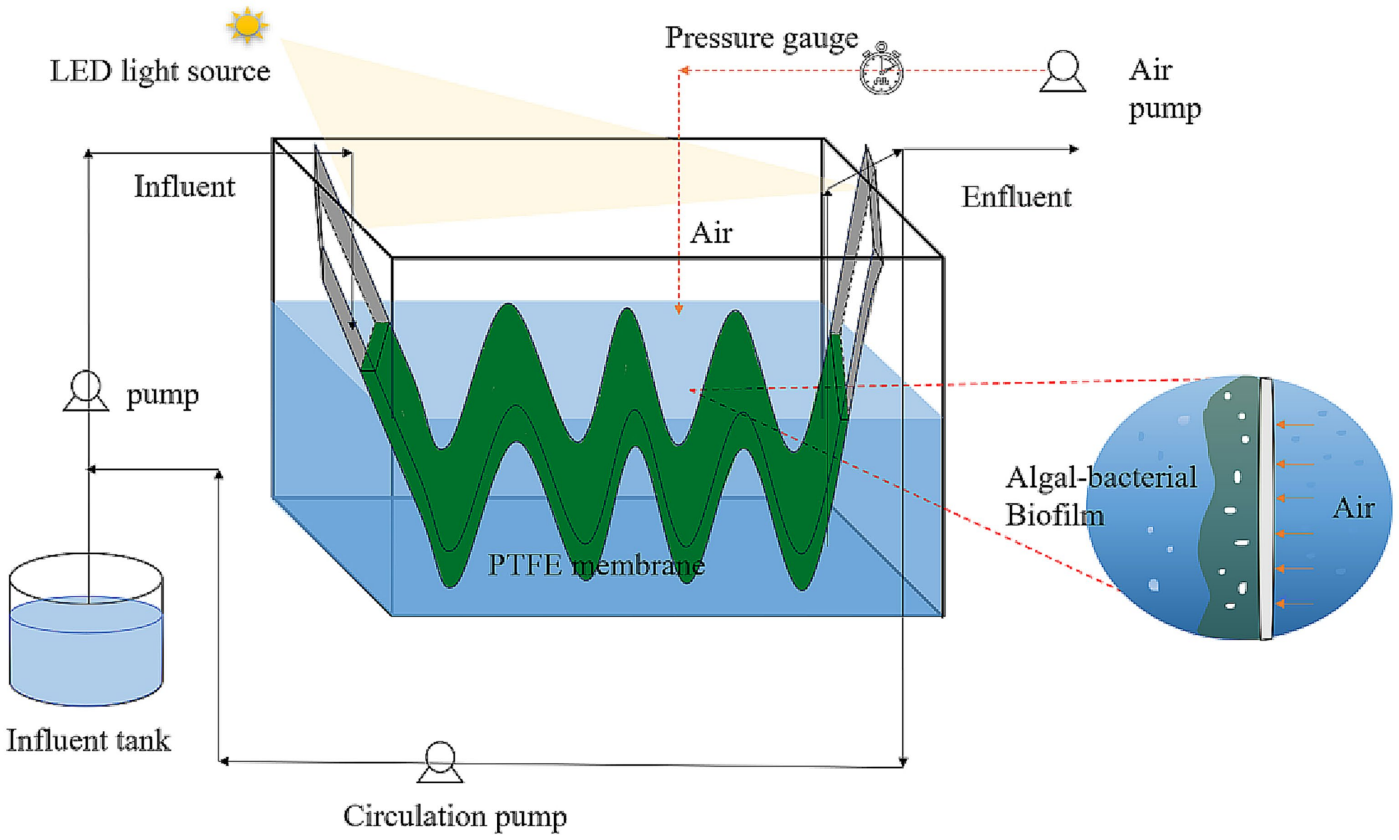
Schematic illustration of the AB-MABR experimental systems.

Two plexiglass MABRs were constructed, each with an effective working volume of 2.8 L. One reactor containing algae was designated as AB-MABR, while the other, which contained only sludge (without algae) was designated as B-MABR (Figure 1). Prior to reactor start-up, the activated sludge and algal suspension were mixed at a biomass ratio of 1:1 and inoculated into the AB-MABR reactor, whereas only activated sludge was introduced into the B-MABR reactor. The initial mixed liquor suspended solids (MLSS) concentration in both reactors was adjusted to approximately 1,500 mg/L. During the subsequent 20-day biofilm development period, all operating conditions were kept identical to ensure a fair comparison between the AB-MABR and B-MABR systems. An external LED light source was installed around the reactors to provide illumination at a light intensity of 5,000 lx. To eliminate light interference, the B-MABR was enclosed with an opaque curtain. The biofilm development period lasted for 20 days under the same operating conditions as those used during the experimental stage. Biofilm maturity was considered to be achieved when the pollutant removal performance became stable and a continuous, well-developed biofilm layer was observed on the membrane surface. After the biofilm reached this stable state, the bulk liquid was discharged and the formal experimental phase was initiated.

Both reactors were equipped with polytetrafluoroethylene (PTFE) hollow-fiber membrane modules, which possessed a total surface area of 1,300 cm^2^ and an average pore diameter of 0.22 μm. The modules were fabricated by bonding flat membrane sheets into a hollow configuration. Oxygen was supplied to the lumens of the hollow fibers via an air pump and diffused outward into the biofilm through the membrane walls. The reactors were operated in continuous-flow mode at a C/N ratio of 4 to simulate typical carbon-limited municipal wastewater and side-stream effluents, both of which feature insufficient biodegradable organic carbon for effective heterotrophic denitrification. The operational parameters were set as follows: a hydraulic retention time (HRT) of 24 h, a circulation pump speed of 80 rpm, and an aeration rate of 2 L/min. The pH was maintained between 7.0 and 8.0, and the temperature was controlled within 25–28 °C throughout the operational period. The biofilm was seeded for 20 days under the same operational conditions as the subsequent experiments. Once matured, the bulk liquid was discharged, marking the start of the formal experimental phase.

### 2.3 Water quality analysis

Wastewater samples were first filtered through a 0.45 μm membrane. The concentrations of COD, TN, NH_4_^+^-N, nitrate nitrogen (NO_3_^−^-N), and nitrite nitrogen (NO_2_^−^-N) were then measured every 2 days according to the standard methods (APHA, 2005). All data were collected during the steady-state period, with all samples analyzed in triplicate.

On the final day of operation, biofilm samples were gently collected from three different points within the same region of the membrane module. A 1 cm^2^ section from each collected biofilm was cut for analysis. EPS were extracted using a thermophysical method (Sun et al., 2017). The protein content in the EPS was quantified by the Lowry method, while the polysaccharide content was determined using the phenol-sulfuric acid method. The sum of protein and polysaccharide concentrations was defined as the total EPS concentration.

### 2.4 Metabolic activity and electron transfer analysis

To evaluate the metabolic activity of the biofilm, the adenosine triphosphate (ATP) content was determined using a commercial ATP Assay Kit (BC0305, Solarbio, Beijing, China) according to the manufacturer’s instructions. Briefly, approximately 0.1 g (wet weight) of biofilm was homogenized in ATP extraction buffer in an ice bath and then centrifuged at 10,000 × g for 10 min at 4 °C. The supernatant was mixed with chloroform to remove interfering substances, and the aqueous phase was collected for ATP determination. Following incubation at 37 °C for 3 min, absorbance was measured at 340 nm using a microplate reader. ATP concentrations were calculated according to the manufacturer’s protocol, and the results were normalized to protein content and expressed as μmol/g protein.

Electron transport system activity (ETSA) was determined using the iodonitrotetrazolium chloride (INT) reduction method. Briefly, 1 mL of biofilm suspension was mixed with 2 mL of 0.2% INT solution and incubated in the dark at 20 °C for 20 min. The reaction was terminated by adding 0.2 mL formaldehyde. After centrifugation at 10,000 × g for 5 min, the pellet was extracted twice with 96% methanol, and the absorbance of the combined extracts was measured at 495 nm using a UV–Vis spectrophotometer. ETSA was calculated according to the method described by Maurines-Carboneill et al. (1998).

Cytochrome c (Cyt-c) content was determined spectrophotometrically following the method of Pérez-Mejías et al. (2019). Biofilm samples were suspended in phosphate-buffered saline (PBS, 0.1 M, pH 7.4) and disrupted by repeated freeze–thaw cycles to extract intracellular Cyt-c. The lysates were centrifuged at 12,000 × g for 15 min at 4 °C, and the resulting supernatants were collected for analysis. The absorbance difference between the reduced and oxidized forms of Cyt-c was measured at 550 and 535 nm using a UV–Vis spectrophotometer. Cyt-c concentrations were calculated according to the Beer–Lambert law using a molar extinction coefficient of 21.1 mM-1 cm-1, normalized to volatile suspended solids (VSS), and expressed as mg g-1 VSS. All measurements were performed in triplicate.

### 2.5 Biofilm morphological and structural characteristics analysis

#### 2.5.1 Fluorescence excitation-emission matrix (EEM) analysis

Three-dimensional excitation-emission matrix (3D-EEM) fluorescence spectroscopy was conducted using a fluorescence spectrophotometer (FP6500, JASCO, Japan). The excitation wavelength (Ex) was scanned from 200 to 450 nm at 5 nm intervals, while the emission wavelength (Em) was scanned from 250 to 550 nm at 2 nm intervals. The slit width was set to 5 nm, and the scanning speed was maintained at 2000 nm/min.

#### 2.5.2 Morphology analysis

Samples were fixed in 2.5% (v/v) glutaraldehyde solution at 4 °C for 24 h, followed by rinsing with 0.1 mol/L phosphate buffer solution (PBS). The samples were then dehydrated through a graded ethanol series, replaced with tert-butanol, and subjected to freeze-drying. After sputter-coating with gold, biofilm morphology was observed using scanning electron microscopy (SEM), and elemental composition of selected regions was analyzed using energy-dispersive X-ray spectroscopy (EDS).

For confocal laser scanning microscopy (CLSM) analysis, biofilm samples were stained with specific fluorescent probes targeting proteins, polysaccharides, and total cells, respectively. The stained samples were then visualized using CLSM. The spatial distribution and relative abundance of biofilm components were quantified by integrated optical density (IOD) analysis using Image-Pro Plus 6.0 software.

### 2.6 Microbial function

At the end of the experiment, biofilm samples were collected from both reactors for metagenomic analysis. Total genomic deoxyribonucleic acid (DNA) was extracted using the E.Z.N.A.® Soil DNA Kit (Omega Bio-tek, USA) according to the manufacturer’s instructions. DNA integrity was verified by 1% agarose gel electrophoresis, and DNA concentration and purity were determined using a NanoDrop 2000 UV–Vis spectrophotometer (Thermo Scientific, USA).

Metagenomic libraries were constructed and sequenced on the Illumina HiSeq 2,500 platform (Illumina, USA) using a paired-end strategy (2 × 150 bp). Each sample generated approximately 13 Gb of clean sequencing data, corresponding to an average of 87.8 million clean paired-end reads. Raw sequencing reads were quality-filtered using Trimmomatic (v0.36) to remove adapter sequences, reads containing ambiguous bases, and low-quality reads (Phred score < 20). High-quality reads were assembled *de novo* using MEGAHIT (v1.2.9) with default parameters. Open reading frames (ORFs) were predicted using Prodigal (v2.6.3). Taxonomic annotation was performed against the NCBI non-redundant (NR) database, while functional annotation was conducted against the Kyoto Encyclopedia of Genes and Genomes (KEGG, Release 95.0) database using BLASTP with an E-value cutoff of 1 × 10^−5^. Functional profiles were generated based on annotated KEGG orthologues (KOs) and their corresponding metabolic pathways.

## 3 Results and discussion

### 3.1 Nutrient removal performance

To assess the wastewater treatment performance of the AB-MABR and B-MABR systems, the influent and effluent concentrations of COD, NH_4_^+^-N, and TN were monitored under a C/N ratio of 4. Under this condition, the influent COD concentration was 200.5 mg/L. The effluent COD concentration in the B-MABR (22.54 mg/L) was 46.94% higher than that in the AB-MABR (15.34 mg/L). This result indicated that the COD removal efficiency was improved after inoculating microalgae into the MABR system. Considering the low C/N ratio (C/N = 4) of the influent wastewater, biodegradable organic carbon became a limiting factor for microbial metabolism. The enhanced COD removal in the AB-MABR system was likely attributed to the synergistic interaction between microalgae and bacteria. Photosynthetically produced oxygen supplemented membrane aeration and may have promoted the aerobic oxidation of organic matter, while the algal-bacterial symbiosis enhanced microbial metabolic activity and carbon utilization efficiency (Li et al., 2022). This explanation was further supported by the higher ATP content observed in the AB-MABR system.

**Figure 2:**
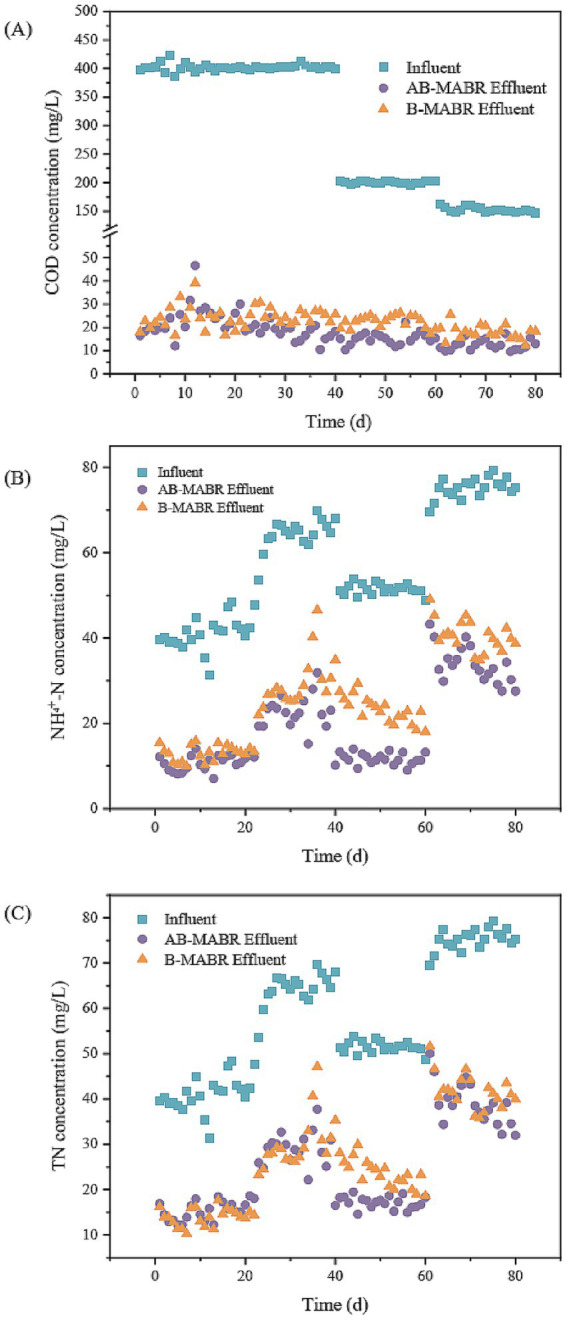
Removal efficiencies of COD **(A)**, NH_4_^+^-N **(B)**, and TN **(C)**.

The AB-MABR system exhibited significantly superior nitrogen removal performance, achieving NH_4_^+^-N and TN removal efficiencies of 77.2 and 66.6%, respectively, compared to only 55.3 and 54.3% in the control B-MABR. These results demonstrate that the AB-MABR’s performance was not constrained by the low carbon availability inherent to the system. The enhanced NH_4_^+^-N removal observed in the AB-MABR system may be partially associated with oxygen generated through microalgal photosynthesis, which could contribute to a more favorable environment for nitrifying microorganisms. Furthermore, denitrification in the B-MABR was strongly limited by the low C/N ratio, resulting in incomplete nitrogen removal and consequently similar NH_4_^+^-N and TN removal efficiencies. The superior TN removal in the AB-MABR is attributed to the dual benefit provided by the algae-bacteria consortium: enabling the conventional nitrification–denitrification pathway and enhancing overall nitrogen removal through direct algal assimilation of various nitrogen species (ammonia, nitrate, and nitrite) (Figure 2).

### 3.2 Microbial activity and electron transfer characteristics

**Figure 3:**
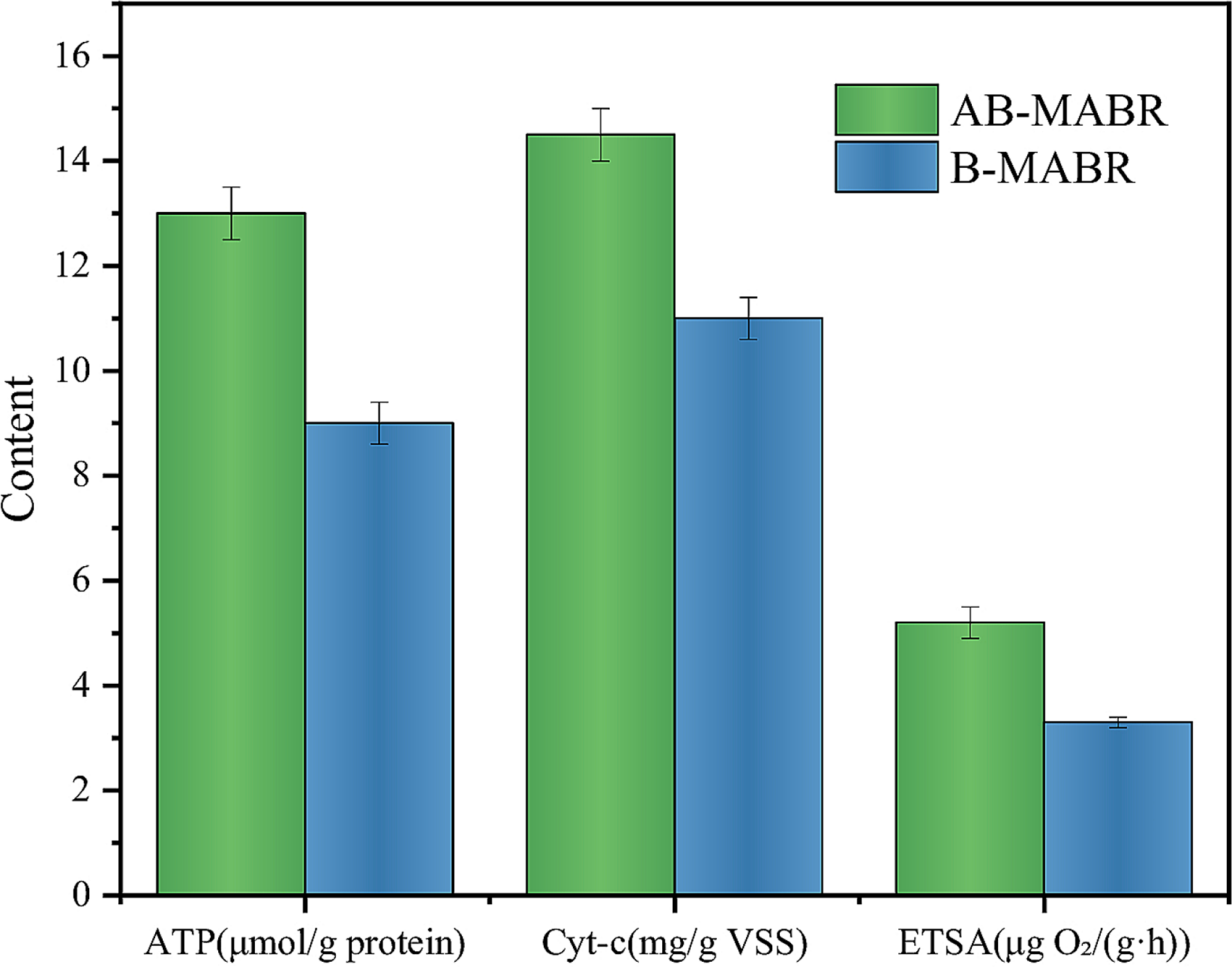
Biofilm ATP, Cyt-c and ETSA concentrations.

ATP is a key indicator of microbial metabolic activity, as it directly reflects intracellular energy availability and physiological status. Therefore, ATP concentrations were measured to evaluate the metabolic activity of the AB-MABR and B-MABR systems. As shown in Figure 3, the ATP content in the AB-MABR system reached 13.5 μmol/g protein, which was significantly higher than that in the B-MABR system (9.3 μmol/g protein). This result indicates that the incorporation of algae enhanced microbial metabolic activity, likely by promoting synergistic interactions between algae and bacteria that improved energy production efficiency. This enhanced metabolic activity is directly linked to improved pollutant removal performance. ATP serves as the primary energy currency for microbial metabolism, supporting key biochemical processes such as organic matter degradation and nitrogen transformation (Mu et al., 2024). Higher ATP availability can accelerate enzymatic reactions involved in carbon oxidation, thereby enhancing COD removal. Meanwhile, sufficient energy supply is essential for energy-intensive processes such as nitrification and denitrification, promoting ammonia oxidation and nitrate reduction (Dou et al., 2023). Therefore, the elevated ATP levels in the AB-MABR system likely contributed to more efficient carbon utilization and nitrogen removal, resulting in the superior treatment performance observed. Furthermore, higher ATP levels are generally associated with increased EPS production, which plays a crucial role in maintaining biofilm stability and supporting microbial metabolism (Lai et al., 2018). In addition, the elevated ATP content in the AB-MABR system suggests enhanced EPS secretion, which may contribute to providing additional substrates for heterotrophic processes, ultimately facilitating nitrogen removal.

Since ATP generation is driven by electron transport processes, electron transfer characteristics were further investigated to elucidate the underlying mechanisms. The ETSA and Cyt-c content were used as indicators of electron transfer intensity and respiratory activity, respectively (Maurines-Carboneill et al., 1998; Pérez-Mejías et al., 2019). As shown in Figure 3, the ETSA and Cyt-c content in the AB-MABR system reached 5.2 μg O_2_/(g·h) and 14.5 mg/g VSS, respectively, which were significantly higher than those in the B-MABR system (3.3 μg O_2_/(g·h) and 11 mg/g VSS). Such improvements in ETSA and Cyt-c levels imply an augmented microbial electron transport chain activity in the AB-MABR system, which accelerated electron transfer and translocation, ultimately boosting the system’s electron flux and comprehensive metabolic viability. The elevated electron transfer activity in the AB-MABR system indicates intensified redox reactions within the biofilm, which is particularly beneficial under low C/N conditions where electron donors are limited. Enhanced electron transfer may contribute to improving electron utilization and allocation within the denitrification pathway, thereby facilitating the reduction of nitrate and nitrite under carbon-limited conditions (He et al., 2025). Moreover, the higher Cyt-c content in the AB-MABR system suggests more active respiratory processes and a more efficient intracellular electron transport chain, which supports coordinated nitrification–denitrification. In contrast, the lower Cyt-c levels observed in the B-MABR system may indicate restricted electron flow under carbon-limited conditions, leading to insufficient electron supply for denitrification and ultimately constraining nitrogen removal efficiency.

### 3.3 EPS characterization

#### 3.3.1 EPS composition analysis

The EPS content in the AB-MABR system reached 76.96 mg/g VSS, which was significantly higher than that in the B-MABR system (57.43 mg/g VSS), indicating that the introduction of microalgae markedly promoted EPS production. EPS is mainly composed of polysaccharides, proteins, nucleic acids, and lipids. These metabolites not only provide additional carbon sources for bacteria but also stimulate microbial activity and ATP synthesis, thereby enhancing nutrient removal. Among these components, polysaccharides and proteins are particularly important for microbial aggregation, cohesion, and adhesion, contributing to the formation of a compact and stratified biofilm structure with stable thickness (Wang et al., 2020; Cheah and Chan, 2022). Therefore, the elevated EPS content in the AB-MABR system likely enhanced biofilm integrity and structural stability compared with the B-MABR system. In addition, the dense EPS matrix may provide a protective microenvironment for microorganisms, improving their resistance to environmental fluctuations and maintaining stable biological activity.

Furthermore, EPS plays multiple functional roles in nitrogen removal. Organic substances within EPS, especially polysaccharides and proteins, may serve as supplementary carbon and energy sources for microorganisms, thereby alleviating carbon limitation under low C/N ratio conditions (Sheng et al., 2010; Huang et al., 2022). Meanwhile, abundant functional groups in EPS, such as carboxyl, hydroxyl, and amide groups, may facilitate the adsorption and retention of nitrogenous compounds (e.g., NH_4_^+^, NO_2_^−^, and NO_3_^−^) within the biofilm matrix, which enhances substrate enrichment and mass transfer efficiency (Huang et al., 2022). Previous studies have also suggested that EPS may participate in extracellular electron transfer by acting as an electron-transfer mediator, thereby potentially facilitating denitrification activity (Sathishkumar et al., 2020). Consequently, the superior nitrogen removal performance observed in the AB-MABR system may be closely associated with its elevated EPS content. The enhanced EPS production not only strengthened biofilm stability and improved substrate transfer but also reinforced the synergistic interactions between microalgae and bacteria. This may establish a positive feedback mechanism in which AB-MABR stimulates EPS synthesis, while the increased EPS further promotes microbial cooperation and nitrogen removal efficiency.

#### 3.3.2 EEM fluorescence analysis of EPS

**Figure 4:**
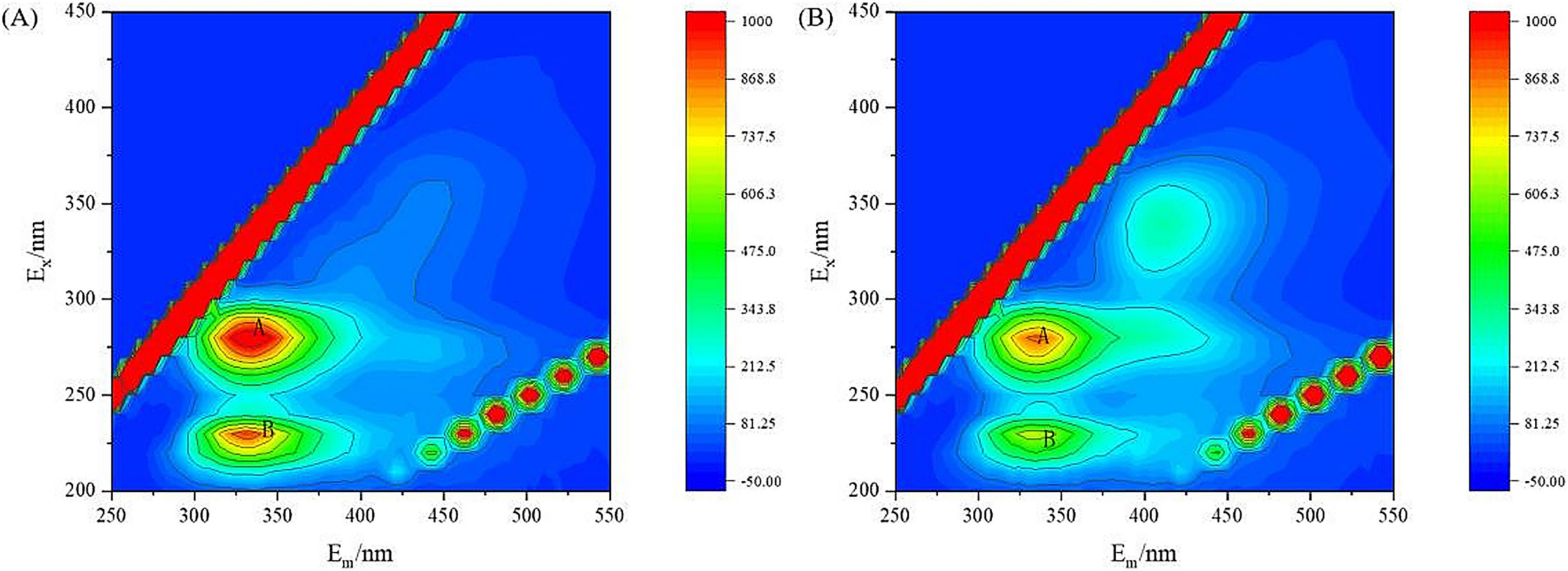
EEM spectra: **(A)** for the microalgae-bacteria MABR system and **(B)** for the sludge-MABR system.

EEM fluorescence spectroscopy is an effective technique for characterizing the composition and transformation of dissolved organic matter and EPS in biological wastewater treatment systems. By scanning samples over a range of excitation and emission wavelengths, EEM generates a three-dimensional fluorescence profile in which the x-axis represents the emission wavelength, the y-axis represents the excitation wavelength, and the fluorescence intensity is reflected by color distribution. This method enables the identification of different fluorescent components in EPS and provides insights into microbial metabolic activity, organic matter transformation, and pollutant removal performance (Sheng and Yu, 2006). The three-dimensional EEM spectra of EPS extracted from the AB-MABR and B-MABR systems are shown in Figure 4. As illustrated in Figure 4, both the B-MABR and AB-MABR systems exhibited distinct fluorescence peaks in the regions of Ex ≈ 230 nm/Em ≈ 340 nm and Ex ≈ 280 nm/Em ≈ 330 nm, which are typically attributed to tryptophan-like and tyrosine-like protein substances, respectively. These protein-like substances are important constituents of EPS and play a critical role in maintaining biofilm structural integrity. Due to their hydrophobic properties, tryptophan-like and tyrosine-like proteins can enhance intermolecular interactions among EPS components, thereby strengthening microbial adhesion, aggregation, and biofilm stability (He et al., 2024). In addition, tryptophan-containing substances have been reported to facilitate extracellular electron transfer (Shih et al., 2008), which may enhance microbial metabolic cooperation and denitrification activity. Furthermore, these protein-like substances can serve as readily biodegradable endogenous carbon sources (Fan et al., 2021), which is particularly beneficial for nitrogen removal under low C/N ratio conditions.

Compared with the B-MABR system, the fluorescence intensities of the two protein-like peaks in the AB-MABR system increased by 33.8 and 26.6%, respectively, indicating that the introduction of microalgae significantly promoted the accumulation of proteinaceous EPS components. This enhancement suggests stronger microbial metabolic activity and improved biofilm formation in the AB-MABR system. The higher abundance of protein-like substances may further reinforce microbial attachment and facilitate electron transfer processes, thereby contributing to enhanced nitrogen removal performance.

In addition to protein-like peaks, both systems displayed humic-like fluorescence signals in the region of Ex ≈ 330–350 nm and Em ≈ 420–450 nm. Notably, the fluorescence intensity of this region was markedly higher in the AB-MABR system than in the B-MABR system, suggesting a greater degree of organic matter transformation and humification. Humic-like substances are generally considered to be more stable metabolic byproducts formed during microbial degradation and transformation processes. Their higher accumulation in the AB-MABR system implies that the microalgae-bacteria symbiotic relationship enhanced the conversion and utilization of organic substrates, leading to more active internal carbon cycling within the biofilm.

Overall, the EEM results demonstrate that the AB-MABR system contained higher levels of both protein-like and humic-like substances in EPS compared with the control system. The enrichment of these fluorescent components not only enhanced biofilm stability and microbial activity but also improved endogenous carbon supply and electron transfer capacity, thereby facilitating denitrification and pollutant removal. These findings are consistent with the previously observed superior TN removal efficiency of the AB-MABR system and further confirm the important role of microalgae-induced EPS enhancement in promoting stable and efficient nitrogen removal. Specifically, the increased abundance of protein-like substances, particularly tryptophan-like compounds, may enhance extracellular electron transfer by serving as redox-active components within the EPS matrix. This interpretation is consistent with the elevated ETSA and Cyt-c levels observed in the AB-MABR system, indicating strengthened electron transport activity. Moreover, humic-like substances are known to possess electron-shuttling properties and can function as transient electron carriers between electron donors and denitrifying microorganisms (Song et al., 2025). Therefore, the accumulation of humic-like substances may improve electron utilization efficiency and facilitate nitrate and nitrite reduction under carbon-limited conditions. These findings suggest that microalgae-induced EPS production not only strengthened the biofilm structure but also promoted denitrification through enhanced electron transfer process.

### 3.4 Biofilm structure and morphology

#### 3.4.1 Surface morphology analysis

**Figure 5:**
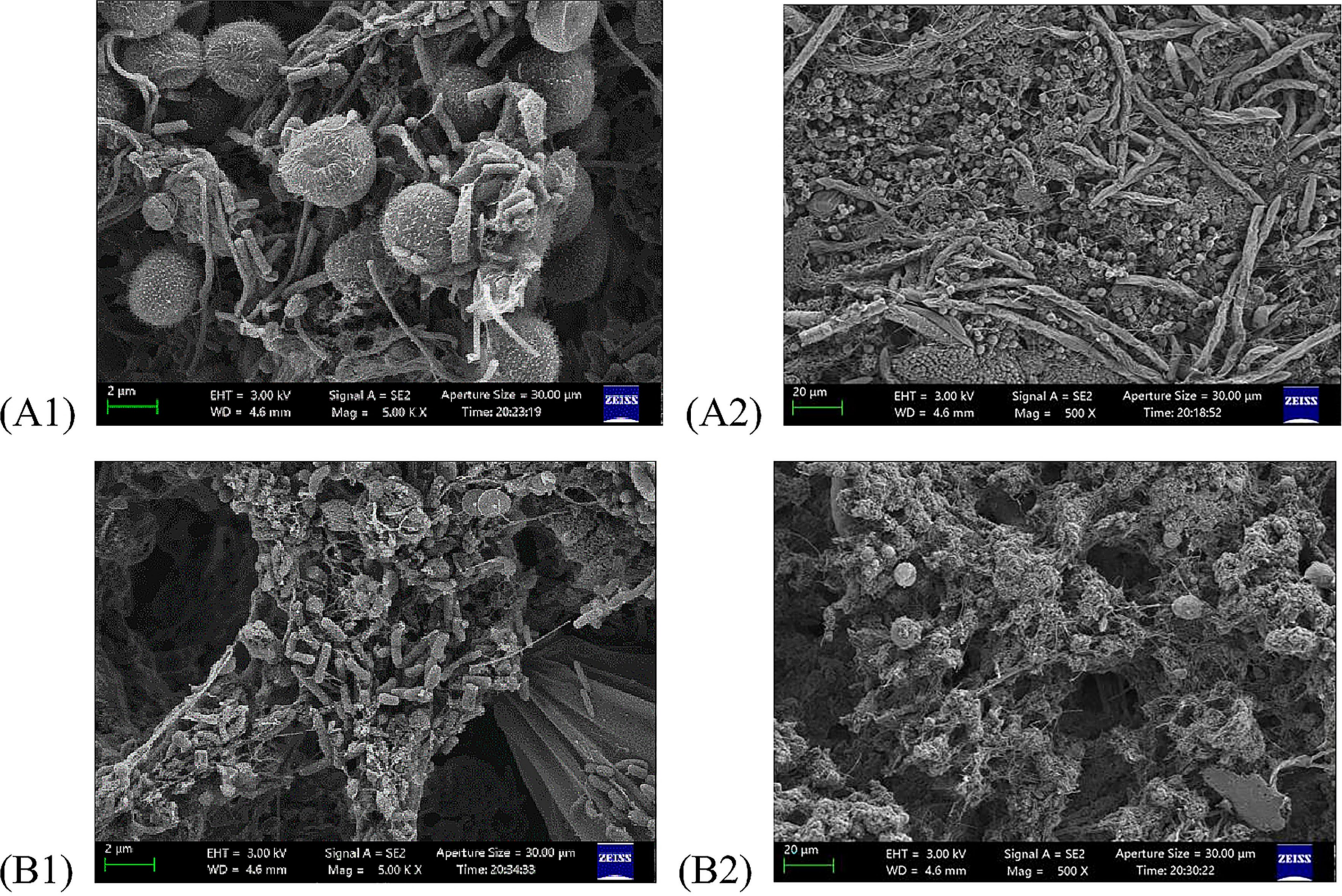
SEM observation results of the biofilm: **(A)** and **(B)** represent algae-bacteria biofilm and the sludge biofilm respectively; (1) and (2) are the magnifications of 2 μm and 20 μm, respectively.

As shown in Figure 5A1, spherical algal cells and rod-shaped bacteria were closely aggregated and interconnected, forming a dense reticular network structure. This interaction was not limited to simple physical attachment; instead, the microalgae and bacteria were embedded within an EPS-rich matrix that tightly integrated the microbial community into a stable symbiotic consortium. The EPS matrix acted as a bridging and protective layer, enhancing microbial adhesion and cohesion while maintaining the integrity of the biofilm structure. This compact and interconnected structure is conducive to maintaining close physical associations between microalgae and bacteria, which may facilitate efficient nutrient exchange and synergistic metabolism (Wang et al., 2022). However, direct evidence for such metabolic interactions, such as nutrient transfer or signaling molecule exchange, still requires further investigation using complementary techniques, such as isotope tracing and microsensor analyses. In contrast, Figure 5B1 shows that the biofilm in the B-MABR system was mainly composed of bacterial flocs with a relatively loose and discontinuous structure. The biofilm lacked the characteristic three-dimensional interwoven network observed in the AB-MABR system. Microorganisms appeared to aggregate irregularly, with visible gaps and less compact organization, indicating weaker structural stability and lower resistance to external shear stress.

The SEM observations shown in Figure 5 further confirmed these structural differences. In Figure 5A2, algal filaments and bacterial cells formed a highly intertwined reticular framework, clearly demonstrating the establishment of a stable microalgae-bacteria symbiotic consortium. The algal filaments acted as a natural scaffold that provided attachment sites and structural support for bacterial colonization, while bacterial cells adhered to and twined around the filament surfaces. Meanwhile, EPS filled the spaces among microorganisms, further reinforcing the three-dimensional network architecture. This highly organized structure resulted in a dense, uniform, and compact biofilm with enhanced spatial stability. By comparison, Figure 5B2 illustrates the sludge biofilm formed in the control system, where microorganisms were unevenly distributed and obvious voids were present throughout the structure. Without the supporting effect of algal filaments, the sludge biofilm lacked a stable framework and exhibited weaker compactness and structural integrity. The relatively loose architecture may reduce microbial retention capacity and increase the susceptibility of the biofilm to detachment during reactor operation.

The superior structural characteristics of the AB-MABR biofilm can be attributed to three major factors. First, the higher EPS secretion in the AB-MABR system significantly enhanced microbial adhesion and aggregation, which is essential for the establishment and stabilization of the microalgae-bacteria symbiotic consortium. Second, algal filaments functioned as a biological scaffold, providing mechanical support and promoting the formation of a denser biofilm structure. Third, the EPS matrix formed a continuous reticular network that strengthened the interactions among microorganisms and improved the attachment between the biofilm and membrane surface.

Overall, the incorporation of microalgae played a crucial role in shaping biofilm morphology and stability. The compact and interconnected microalgae–bacteria biofilm facilitated substrate diffusion and mass transfer, thereby enhancing nitrogen uptake, transformation, and overall pollutant removal performance. In contrast, the relatively loose sludge biofilm in the B-MABR system was more vulnerable to structural disruption and biofilm detachment, which could negatively affect reactor stability and long-term operational efficiency.

#### 3.4.2 Three-dimensional structure analysis

To further investigate the spatial structure and compositional distribution of microorganisms within the biofilm, CLSM was employed. CLSM is an advanced optical imaging technique that uses a laser light source combined with a confocal pinhole system to obtain high-resolution two-dimensional images of biofilm surfaces. Through continuous layer-by-layer scanning along the z-axis, a three-dimensional reconstruction of the biofilm can be generated, enabling detailed visualization of the biofilm’s architecture, thickness, and component distribution (Mhade and Kaushik, 2023). In this study, different fluorescent dyes were used to selectively stain specific biofilm constituents, including extracellular proteins (green), polysaccharides (red), and total microbial cells (blue).

**Figure 6:**
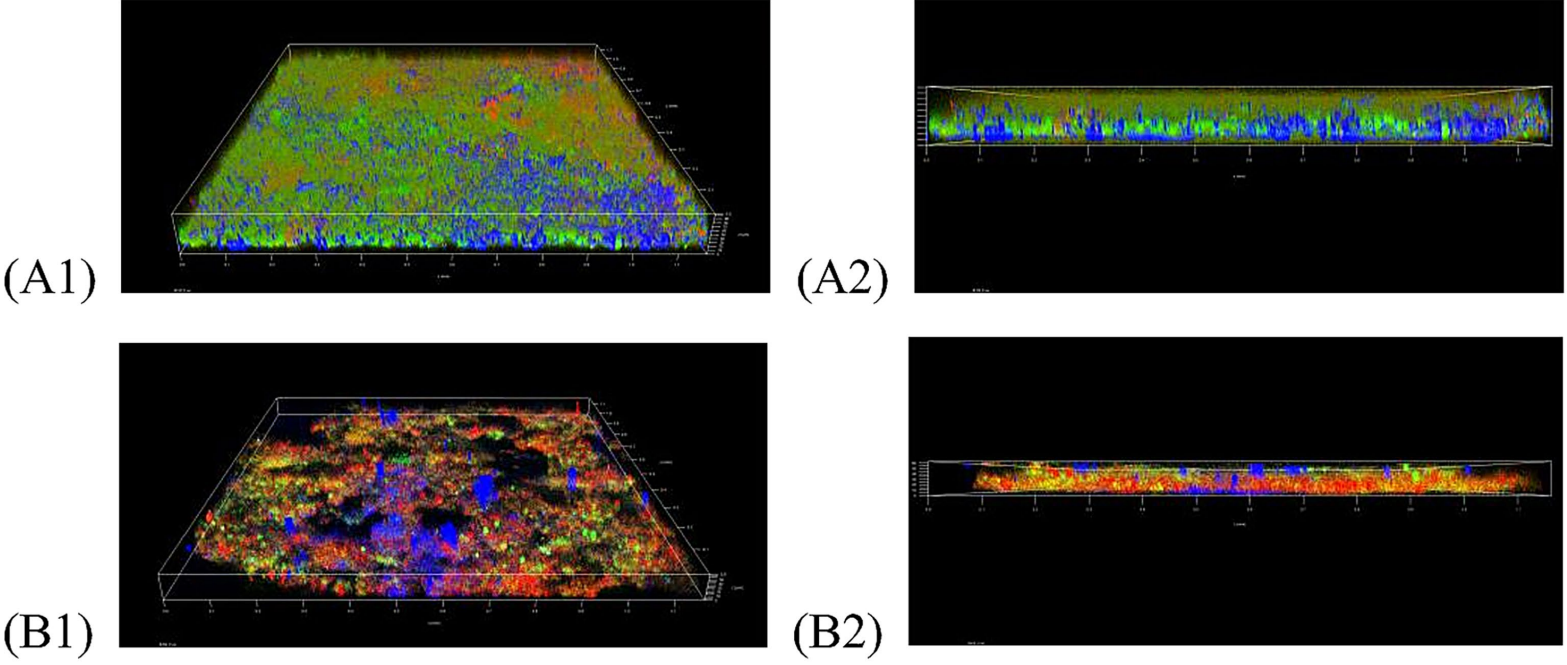
CLSM observation of biofilms: **(A)** and **(B)** represent the algae-bacteria biofilm and sludge biofilm, respectively; (1) and (2) are the overall planar image and the three-dimensional image, respectively.

As shown in Figure 6, the algal-bacterial biofilm in the AB-MABR system exhibited substantially greater thickness, stronger fluorescence intensity, and a more uniform spatial distribution than the sludge biofilm in the B-MABR system. The algal-bacterial biofilm formed a dense and highly stratified three-dimensional structure with clear spatial organization, whereas the sludge biofilm appeared relatively loose, discontinuous, and poorly layered. The thinner structure observed in the sludge biofilm may be attributed to the carbon and oxygen limitations in the control system, which suppressed microbial growth and restricted biofilm development. In contrast, the presence of microalgae in the AB-MABR system likely alleviated these limitations by supplying photosynthetically derived oxygen and organic metabolites, thereby supporting microbial proliferation and stable biofilm formation.

The component distribution within the two biofilms also differed significantly. The algal-bacterial biofilm exhibited stronger extracellular protein-associated fluorescence, indicating the enrichment of proteinaceous EPS components and the formation of a robust cellular framework. Extracellular proteins are important for microbial adhesion, aggregation, and structural stability, and their abundance suggests enhanced microbial interactions and stronger biofilm cohesion. By comparison, the sludge biofilm was characterized by relatively stronger polysaccharide-associated signals. This phenomenon may be related to the adaptive response of heterotrophic bacteria under unfavorable environmental conditions, where extracellular polysaccharides are secreted to protect cells against nutrient stress and environmental fluctuations. In addition, the stronger total-cell fluorescence observed in the algal-bacterial biofilm indicated a substantially higher biomass and microbial density than in the sludge biofilm. Notably, most of the total-cell signals were concentrated in the lower and middle layers of the algal-bacterial biofilm, suggesting the establishment of a compact and active microbial community throughout the biofilm depth. Due to the greater thickness and compactness of the algal-bacterial biofilm, partial attenuation of fluorescence signals occurred during laser penetration, which further indirectly confirmed the development of a dense multilayer structure. These findings demonstrate that the synergistic interactions between microalgae and bacteria effectively enhanced biomass accumulation and maintained high microbial activity within the reactor.

**Figure 7:**
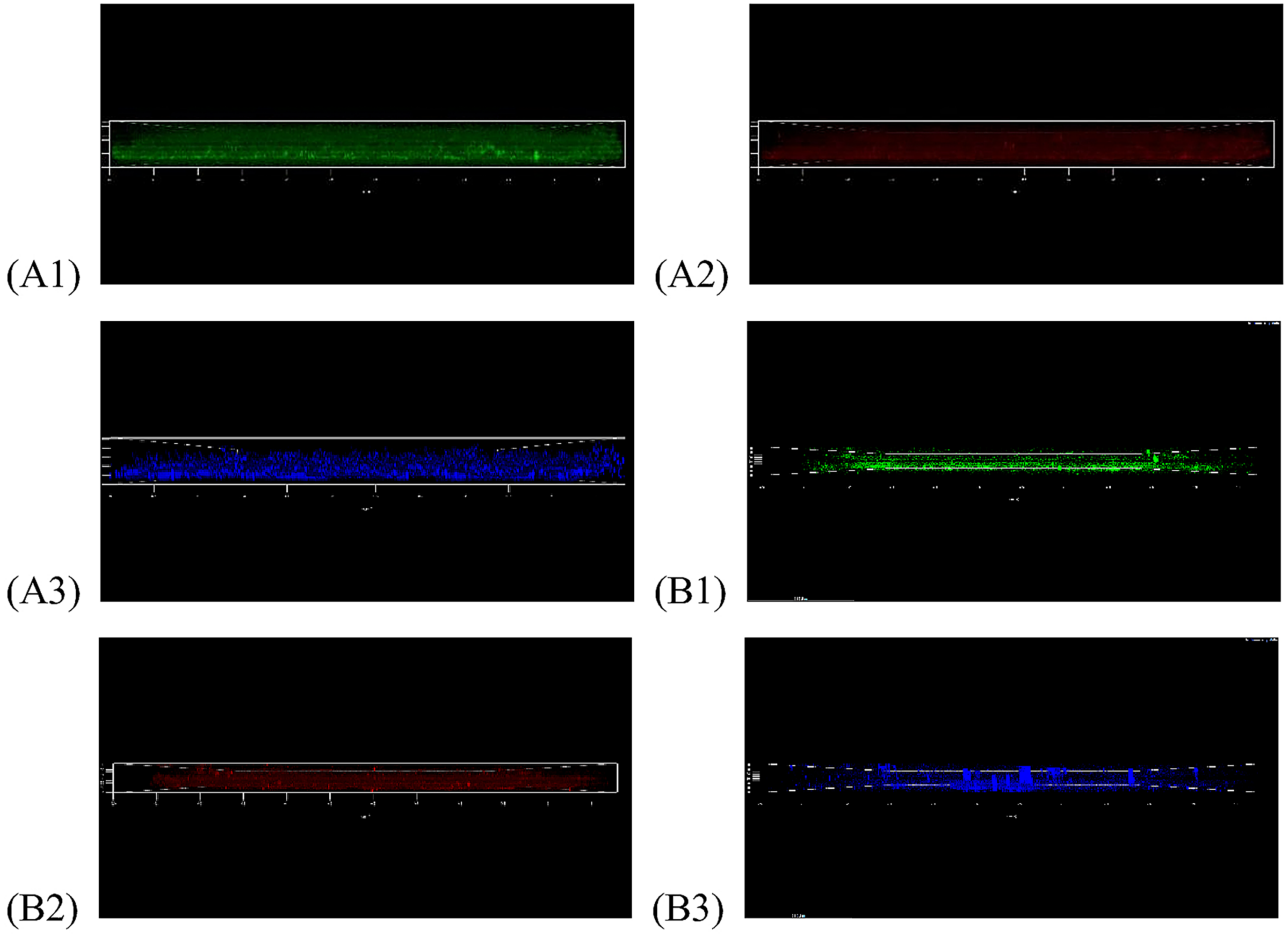
CLSM observation of biofilms: **(A)** and **(B)** represent the algae-bacteria biofilm and sludge biofilm, respectively; (1), (2), and (3) represent extracellular protein, polysaccharides, and total cells, respectively.

To further characterize the three-dimensional architecture and component distribution of the biofilms, cross-sectional CLSM images of extracellular proteins (EP), polysaccharides (PS), and total cells were quantitatively analyzed for both systems as shown in Figure 7. Because fluorescence intensity is positively correlated with the concentration of fluorescent substances, the results revealed that the algal-bacterial biofilm contained significantly higher extracellular protein levels and viable cell abundance than the sludge biofilm, while the polysaccharide content showed relatively smaller differences between the two systems. These results indicate that the establishment of microalgae-bacteria symbiosis promoted protein synthesis and supported the retention of a larger active microbial population, thereby enhancing microbial metabolic activity and pollutant degradation capacity.

Overall, the CLSM results clearly demonstrate that the algal-bacterial biofilm possessed a denser and more stable three-dimensional structure, enriched extracellular proteins, and higher microbial biomass compared with the sludge biofilm. These structural and compositional advantages provide an important molecular and spatial basis for the superior pollutant removal performance of the AB-MABR system under the low C/N ratio condition (C/N = 4). Nevertheless, the relationships among biofilm structure, metabolic cooperation, and treatment performance inferred in this study remain largely speculative. Further studies employing direct functional approaches, such as transcriptomic analysis and isotope labeling, are needed to verify these mechanisms and establish causal links.

### 3.5 Metagenomic analysis

#### 3.5.1 Community structure

**Figure 8:**
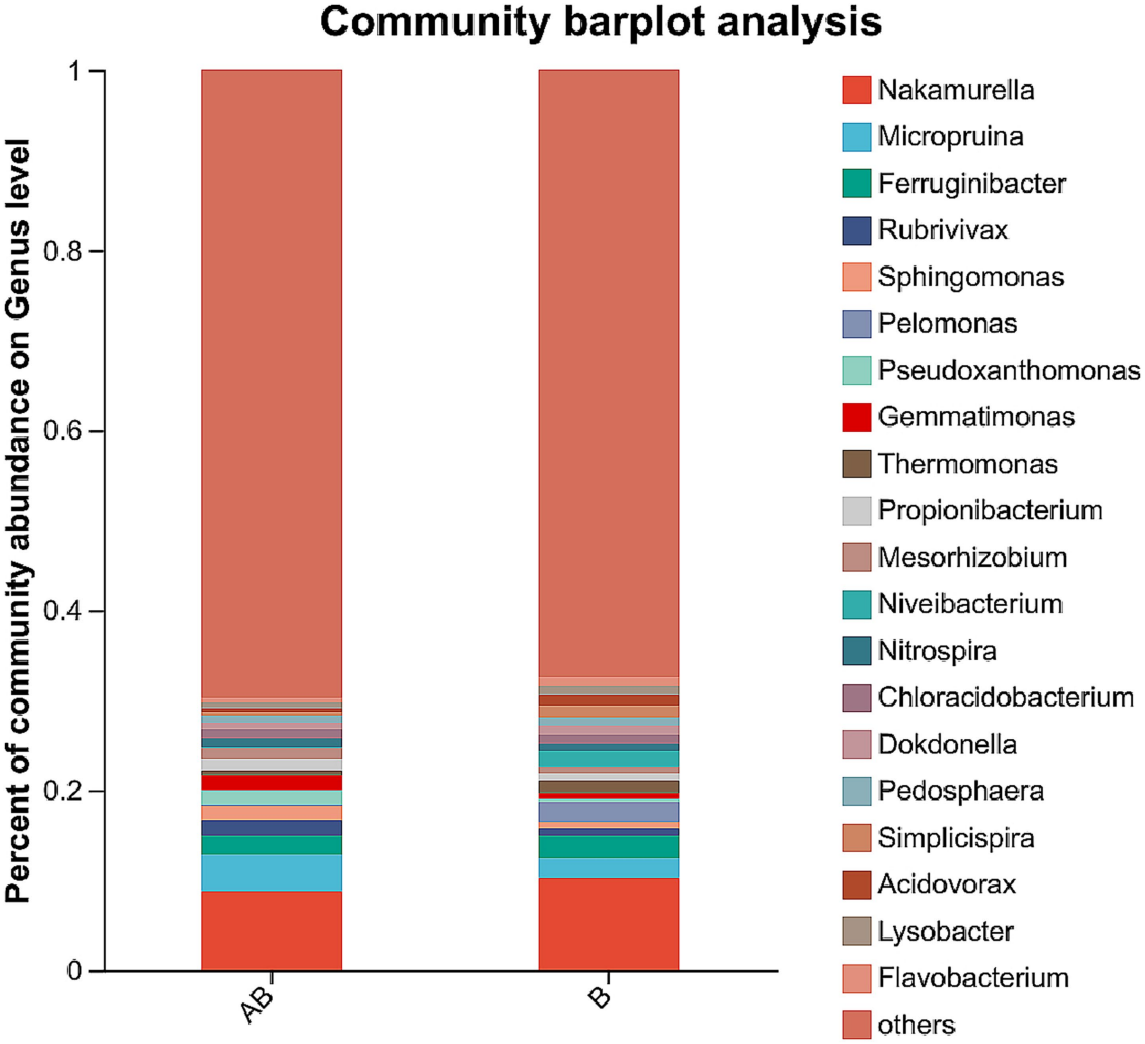
Bacterial community composition at the and genes level in AB-MABR and B-MABR system.

To further elucidate the microbial mechanisms underlying the enhanced nitrogen removal in the AB-MABR system, the bacterial community compositions at the genus levels were analyzed (Figure 8).

At the genus level, microalgal incorporation resulted in pronounced shifts in several functional bacterial populations (Figure 8). Although *Nakamurella* remained the dominant genus in both reactors, multiple genera involved in nitrogen transformation, extracellular electron transfer, and biofilm formation were enriched in the AB-MABR. The relative abundance of *Nitrospira*, a representative nitrite-oxidizing bacterium, increased from 0.86 to 1.00%, which agreed well with the higher abundance of hao and nxrA as well as the improved NH_4_^+^-N removal efficiency. In addition, the abundance of *Rubrivivax* increased from 0.79 to 1.71%. This genus has been widely reported to participate in denitrification and photoheterotrophic metabolism, making it particularly important in algae-bacteria symbiotic systems (Ren et al., 2022).

Several other functional genera, including *Sphingomonas*, *Pseudoxanthomonas*, *Gemmatimonas*, and *Mesorhizobium*, were also substantially enriched in the AB-MABR. These microorganisms have been associated with EPS production, degradation of organic substrates, extracellular electron transfer, and denitrification (Gao et al., 2020). Their enrichment was highly consistent with the increased EPS production, enhanced ETSA, elevated Cyt-c content, and improved TN removal observed in the AB-MABR. Collectively, these results suggest that microalgal incorporation promoted the establishment of a metabolically more active and functionally cooperative bacterial community, thereby enhancing nitrogen conversion under carbon-limited conditions.

Overall, taxonomic profiling demonstrated that microalgal integration did not alter the dominant bacterial phyla, but selectively enriched functional microorganisms associated with nitrification, denitrification, biofilm formation, and electron transfer. These community shifts aligned well with the enrichment of functional genes related to nitrogen metabolism and the superior nitrogen removal performance of the AB-MABR, thereby providing robust microbial ecological insights into the enhanced synergy between microalgae and bacteria under low C/N conditions.

#### 3.5.2 Functional gene expression

To comprehensively elucidate the microbial functional potential and nitrogen transformation mechanisms in different systems, metagenomic analysis was conducted for the AB-MABR and B-MABR systems under the low carbon-to-nitrogen ratio condition (C/N = 4). Metagenomics enables the characterization of microbial metabolic functions by analyzing the abundance and distribution of functional genes and metabolic pathways within microbial communities. To further investigate the mechanisms underlying nitrogen removal, the absolute abundance of specific KEGG metabolic pathways was analyzed.

Both the AB-MABR and B-MABR systems exhibited similar dominant metabolic pathways, mainly including Metabolic pathways, Biosynthesis of secondary metabolites, and Microbial metabolism in diverse environments. Although the overall functional distribution patterns were similar between the two systems, the abundance of these pathways was consistently higher in the AB-MABR system, indicating that the incorporation of microalgae enhanced the metabolic activity and functional potential of the biofilm microbial community. Among these pathways, Metabolic pathways showed the highest abundance in both systems, reaching 301200.8058 in the AB-MABR system and 282445.9085 in the B-MABR system. This category includes essential biochemical processes associated with energy production, nutrient transformation, and biosynthesis of cellular components. The higher abundance observed in the AB-MABR system suggests that microorganisms within the algal-bacterial biofilm possessed stronger metabolic activity and greater energy turnover capacity. Such enhanced metabolic potential likely maintained high microbial activity and promoted efficient nitrogen transformation under low C/N conditions. In addition, oxygen and photosynthetically derived organic metabolites released by microalgae may further stimulate microbial metabolism and support the physiological activity of the biofilm community.

The pathway related to biosynthesis of secondary metabolites also exhibited higher abundance in the AB-MABR system (126169.1726) than in the B-MABR system (115742.8253). Secondary metabolites are typically synthesized by microorganisms in response to environmental stress and ecological competition and play important roles in microbial communication, environmental adaptation, and cooperative interactions (Shibl et al., 2020). The enrichment of this pathway indicates that the microbial community in the algal-bacterial biofilm possessed stronger adaptability and ecological competitiveness. Enhanced synthesis of secondary metabolites may facilitate microbial interactions and stabilize the biofilm community, thereby contributing to improved denitrification and pollutant removal performance. Similarly, the abundance of microbial metabolism in diverse environments was higher in the AB-MABR system (87065.37228) than in the B-MABR system (82079.20542). This pathway reflects the metabolic flexibility and environmental adaptability of microorganisms under changing operational conditions. The higher abundance observed in the AB-MABR system suggests that the algal-bacterial consortium possessed stronger ecological resilience and could maintain stable metabolic activity even under carbon-limited conditions. Such metabolic versatility is beneficial for sustaining continuous nitrogen conversion and long-term reactor stability (Geng et al., 2024).

Overall, the metagenomic results demonstrated that the AB-MABR system harbored a more metabolically active and functionally adaptable microbial community than the conventional sludge biofilm system. The enrichment of pathways related to energy metabolism, environmental adaptation, and microbial metabolic versatility indicated that the microalgae–bacteria symbiotic biofilm exhibited enhanced functional capacity for nutrient transformation under low C/N conditions. These strengthened metabolic characteristics likely promoted key nitrogen conversion processes, including nitrification, denitrification, and nitrogen assimilation, thereby contributing to the superior nitrogen removal performance observed in the AB-MABR system.

**Figure 9:**
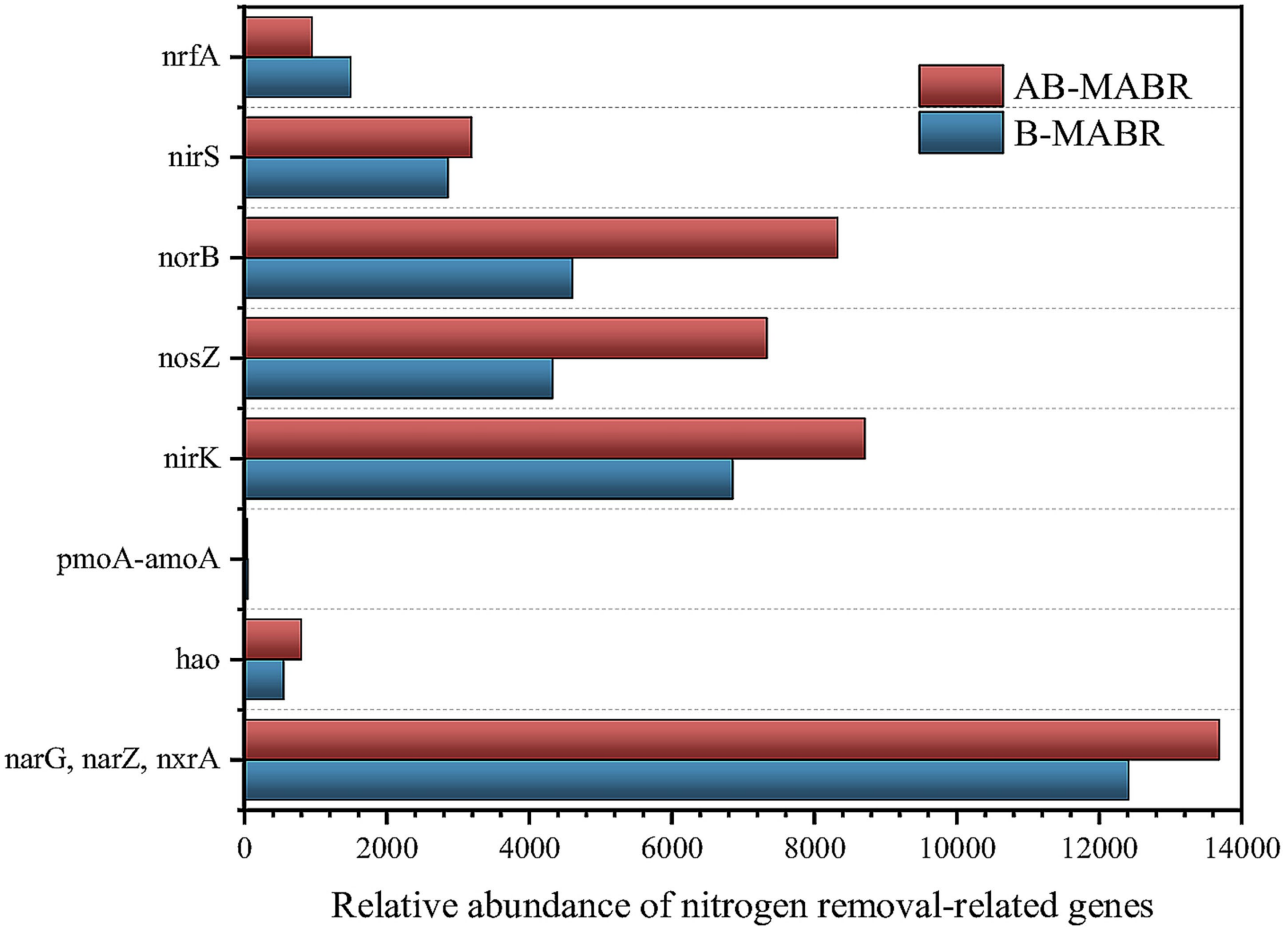
The abundance of nitrogen metabolism genes in the AB-MABR and B-MABR systems.

To investigate the distribution of nitrogen cycling genes in B-MABR and AB-MABR systems, a functional gene set was constructed based on the KEGG database as shown in Figure 9. Results indicated that the overall abundance of these genes was higher in the AB-MABR system than in the B-MABR system, suggesting that algal-bacterial synergy enhanced the functional potential of nitrogen-transforming microorganisms.

During the nitrification process, the relative abundance of the hao gene increased significantly by approximately 46.0% (from 548 in the B-MABR system to 800 in the AB-MABR system), indicating an enhanced hydroxylamine oxidation capacity that facilitated the conversion of ammonium to nitrite (Lei et al., 2021). Meanwhile, the abundance of narG/narZ/nxrA-related genes rose from 12,416 to 13,686, further suggesting an elevated nitrogen transformation potential in the AB-MABR system (Wei et al., 2023). In contrast, the abundance of the pmoA-amoA gene showed negligible variation between the two systems (44 versus 36), implying its relatively limited contribution to the overall nitrogen transformation capacity.

Genes involved in denitrification exhibited a consistently higher abundance in the AB-MABR system. Specifically, the abundance of nirK, nirS, norB, and nosZ increased from 6,856, 2,858, 4,602, and 4,326 to 8,712, 3,186, 8,328, and 7,336, representing increments of 27.1, 11.5, 81.0, and 69.6%, respectively. These genes encode key enzymes responsible for the sequential reduction of nitrite, nitric oxide, and nitrous oxide during denitrification (Wei et al., 2023; Ouyang et al., 2024); thus, their enriched abundance indicated an enhanced denitrification potential in the AB-MABR system (Xia et al., 2024). This enhancement was likely driven by the continuous oxygen release from microalgal photosynthesis, which established a stable DO gradient within the biofilm. Consequently, aerobic nitrification was sustained in the outer biofilm layer, while anoxic microenvironments favorable for denitrifying bacteria were developed in the inner layer. This spatial segregation effectively promoted the coupling of nitrification and denitrification, thereby improving the total nitrogen removal efficiency (Sun et al., 2020).

Additionally, analysis of other nitrogen cycling-related genes revealed that the abundance of the DNRA marker gene nrfA decreased from 1,490 in the B-MABR system to 950 in the AB-MABR system. This trend suggests that the introduction of microalgae, rather than enhancing the DNRA pathway, may have suppressed the reduction of nitrate to ammonium, thereby redirecting more electrons toward denitrification (Wan et al., 2019). Consequently, nitrogen removal in the AB-MABR system was predominantly driven by the nitrification–denitrification pathway rather than DNRA, which is highly beneficial for permanent nitrogen elimination.

Overall, under low carbon-to-nitrogen ratio conditions, the AB-MABR system exhibited higher abundances of key functional genes involved in both nitrification and denitrification, whereas the abundance of the DNRA-related gene decreased. These findings demonstrate that the algal-bacterial synergy optimized the nitrogen metabolic network, strengthened the coupling between nitrification and denitrification, and ultimately enhanced the system’s nitrogen removal performance.

## 4 Conclusion

The incorporation of microalgae significantly improved the performance of the MABR system for nitrogen removal under low C/N conditions. Compared with the conventional B-MABR, the AB-MABR achieved higher COD, NH_4_^+^-N, and TN removal efficiencies, respectively. Mechanistic analyses revealed that microalgae enhanced microbial metabolic activity and electron transfer capacity, as evidenced by the increased ATP, ETSA, and Cyt-c levels. Meanwhile, EPS production, particularly protein-like substances, was markedly promoted, leading to the formation of a denser and more stable biofilm structure with higher biomass and stronger microbial interactions. Metagenomic analysis further demonstrated the enrichment of pathways associated with microbial metabolism, secondary metabolite biosynthesis, and environmental adaptation in the AB-MABR system. These findings provide new insights into the application of algae-bacteria symbiosis in MABR systems for sustainable treatment of low C/N wastewater.

One limitation of this study is that DO concentrations in the bulk liquid and biofilm were not monitored. Therefore, the contribution of photosynthetically generated oxygen to nitrification and nitrogen removal could not be directly quantified. Future studies should combine bulk-liquid DO measurements and oxygen microelectrode profiling to characterize oxygen distribution within algal-bacterial biofilms and further validate the proposed mechanisms.

## Data Availability

The original contributions presented in the study are included in the article/supplementary material, further inquiries can be directed to the corresponding author/s.

## References

[ref3001] APHA A., WEF, (2005). Standard Methods for the Examination of Water and Wastewater, 21st ed. Am. Pub. Health Assoc, Washington, DC.,

[ref1] Bongartz P. Karmainski T. Meyer M. Linkhorst J. Tiso T. Blank L. M. . (2023). A novel membrane stirrer system enables foam-free biosurfactant production. Biotechnol. Bioeng. 120, 1269–1287. doi: 10.1002/bit.28334, 36705321

[ref2] Bose A. Lin R. Rajendran K. O'Shea R. Xia A. Murphy J. (2019). How to optimise photosynthetic biogas upgrading: a perspective on system design and microalgae selection. Biotechnol. Adv. 37:107444. doi: 10.1016/j.biotechadv.2019.107444, 31476422

[ref3] Cheah Y. T. Chan D. J. C. (2022). A methodological review on the characterization of microalgal biofilm and its extracellular polymeric substances. J. Appl. Microbiol. 132, 3490–3514. doi: 10.1111/jam.15455, 35061929

[ref4] Dou Q. Yang J. Peng Y. Zhang L. (2023). Multipathway of nitrogen metabolism revealed by genome-centered metatranscriptomics from pyrite-guided mixotrophic partial denitrification/anammox installations. Environ. Sci. Technol. 57, 21791–21800. doi: 10.1021/acs.est.3c08192, 38079570

[ref5] Fan Z. Zeng W. Meng Q. Liu H. Liu H. Peng Y. (2021). Achieving enhanced biological phosphorus removal utilizing waste activated sludge as sole carbon source and simultaneous sludge reduction in sequencing batch reactor. Sci. Total Environ. 799:149291. doi: 10.1016/j.scitotenv.2021.149291, 34364268

[ref6] Fu X. Hou R. Yang P. Qian S. Feng Z. Chen Z. . (2022). Application of external carbon source in heterotrophic denitrification of domestic sewage: a review. Sci. Total Environ. 817:153061. doi: 10.1016/j.scitotenv.2022.153061, 35026271

[ref7] Gao L. Han F. Zhang X. Liu B. Fan D. Sun X. . (2020). Simultaneous nitrate and dissolved organic matter removal from wastewater treatment plant effluent in a solid-phase denitrification biofilm reactor. Bioresour. Technol. 314:123714. doi: 10.1016/j.biortech.2020.123714, 32593786

[ref8] Geng Y. Xiong Z. Yang L. Lian C. A. Pavlostathis S. G. Qiu Z. . (2024). Bidirectional enhancement of nitrogen removal by indigenous synergetic microalgal–bacterial consortia in harsh low-C/N wastewater. Environ. Sci. Technol. 58, 5394–5404. doi: 10.1021/acs.est.3c10322, 38463002

[ref9] He L. He X. Zhang Y. Fan X. Yang T. Ji X. . (2025). Enhanced dissimilatory nitrate reduction to ammonium and electron transfer mechanisms in bidirectional electron transfer biofilm constructed by iron phthalocyanine. Bioresour. Technol. 426:132381. doi: 10.1016/j.biortech.2025.132381, 40074093

[ref10] He S. Zhao L. Feng L. Zhao W. Liu Y. Hu T. . (2024). Mechanistic insight into the aggregation ability of anammox microorganisms: roles of polarity, composition and molecular structure of extracellular polymeric substances. Water Res. 254:121438. doi: 10.1016/j.watres.2024.121438, 38467096

[ref11] Huang L. Jin Y. Zhou D. Liu L. Huang S. Zhao Y. . (2022). A review of the role of extracellular polymeric substances (EPS) in wastewater treatment systems. Int. J. Environ. Res. Public Health 19:12191. doi: 10.3390/ijerph191912191, 36231490 PMC9566195

[ref12] Lai C. Y. Dong Q. Y. Chen J. X. Zhu Q. S. Yang X. Chen W. D. . (2018). Role of extracellular polymeric substances in a methane based membrane biofilm reactor reducing vanadate. Environ. Sci. Technol. 52, 10680–10688. doi: 10.1021/acs.est.8b02374, 30106284

[ref13] Lei Z. Wang L. Wang J. Yang S. Hou Z. Wang X. C. . (2021). Partial-nitritation of low-strength anaerobic effluent: a moderate-high dissolved oxygen concentration facilitates ammonia-oxidizing bacteria disinhibition and nitrite-oxidizing bacteria suppression. Sci. Total Environ. 770:145337. doi: 10.1016/j.scitotenv.2021.145337, 33736393

[ref14] Li X. Bao D. Zhang Y. Xu W. Zhang C. Yang H. . (2023). Development and application of membrane aerated biofilm reactor (MABR)—a review. Water 15:436. doi: 10.3390/w15030436

[ref15] Li J. Ou R. Liao H. Ma J. Sun L. Jin Q. . (2022). Natural lighting enhancing the algae proliferation and nitrogen removal in membrane-aerated bacterial-algal biofilm reactor. Sci. Total Environ. 851:158063. doi: 10.1016/j.scitotenv.2022.158063, 35981577

[ref16] Liang Z. Zhao Y. Ji H. Li Z. (2025). Algae-bacteria symbiotic biofilm system for low carbon nitrogen removal from municipal wastewater: a review. World J. Microbiol. Biotechnol. 41:218. doi: 10.1007/s11274-025-04405-8, 40555950

[ref17] Maurines-Carboneill C. Pernelle J. J. Morin L. Sachon G. Leblon G. (1998). Relevance of the INT test response as an indicator of ETS activity in monitoring heterotrophic aerobic bacterial populations in activated sludges. Water Res. 32, 1213–1221. doi: 10.1016/s0043-1354(97)00329-1

[ref18] Mhade S. Kaushik K. S. (2023). Tools of the trade: image analysis programs for confocal laser-scanning microscopy studies of biofilms and considerations for their use by experimental researchers. ACS Omega 8, 20163–20177. doi: 10.1021/acsomega.2c07255, 37332792 PMC10268615

[ref19] Mu X. Evans T. D. Zhang F. (2024). ATP biosensor reveals microbial energetic dynamics and facilitates bioproduction. Nat. Commun. 15:5299. doi: 10.1038/s41467-024-49579-1, 38906854 PMC11192931

[ref20] Ouyang L. Zhang W. Chen X. Huang Q. Wang H. Li S. (2024). Insights into the nitrogen removal mechanism of heterotrophic nitrification and aerobic denitrification bacterium *Delfitia* sp. B7. Water 16:3042. doi: 10.3390/w16213042

[ref21] Park J. Craggs R. Shilton A. (2013). Enhancing biomass energy yield from pilot-scale high rate algal ponds with recycling. Water Res. 47, 4422–4432. doi: 10.1016/j.watres.2013.04.001, 23764593

[ref22] Pérez-Mejías G. Guerra-Castellano A. Díaz-Quintana A. de la Rosa M. A. Díaz-Moreno I. (2019). Cytochrome c: surfing off of the mitochondrial membrane on the tops of complexes III and IV. Comput. Struct. Biotechnol. J. 17, 654–660. doi: 10.1016/j.csbj.2019.05.002, 31193759 PMC6542325

[ref23] Ren X. Wang Y. Wan J. Yan Z. Ma Y. Zhang G. . (2022). The nitrogen removal performance and functional bacteria in heterotrophic denitrification and mixotrophic denitrification process. Water 14:3603. doi: 10.3390/w14223603

[ref24] Sathishkumar K. Li Y. Sanganyado E. (2020). Electrochemical behavior of biochar and its effects on microbial nitrate reduction: role of extracellular polymeric substances in extracellular electron transfer. Chem. Eng. J. 395:125077. doi: 10.1016/j.cej.2020.125077

[ref25] Sheng G. P. Yu H. Q. (2006). Characterization of extracellular polymeric substances of aerobic and anaerobic sludge using three-dimensional excitation and emission matrix fluorescence spectroscopy. Water Res. 40, 1233–1239. doi: 10.1016/j.watres.2006.01.023, 16513156

[ref26] Sheng G. P. Yu H. Q. Li X. Y. (2010). Extracellular polymeric substances (EPS) of microbial aggregates in biological wastewater treatment systems: a review. Biotechnol. Adv. 28, 882–894. doi: 10.1016/j.biotechadv.2010.08.001, 20705128

[ref27] Shibl A. A. Isaac A. Ochsenkuhn M. A. Cárdenas A. Fei C. Behringer G. . (2020). Diatom modulation of select bacteria through use of two unique secondary metabolites. Proc. Natl. Acad. Sci. USA 117, 27445–27455. doi: 10.1073/pnas.2012088117, 33067398 PMC7959551

[ref28] Shih C. Museth A. K. Abrahamsson M. Blanco-Rodriguez A. M. di Bilio A. J. Sudhamsu J. . (2008). Tryptophan-accelerated electron flow through proteins. Science 320, 1760–1762. doi: 10.1126/science.1158241, 18583608

[ref29] Song K. Xiao Y. Wang Y. Deng M. Zhou S. Huang Y. . (2025). Electron shuttling promotes denitrification and mitigates nitrous oxide emissions in lakes. Nat. Commun. 16:8564. doi: 10.1038/s41467-025-63601-0, 41022717 PMC12480950

[ref30] Sun J. Dai X. H. Liu Y. W. Peng L. Ni B. J. (2017). Sulfide removal and sulfur production in a membrane aerated biofilm reactor: model evaluation. Chem. Eng. J. 309, 454–462. doi: 10.1016/j.cej.2016.09.146

[ref31] Sun Z. Li M. Wang G. Yan X. Li Y. Lan M. . (2020). Enhanced carbon and nitrogen removal in an integrated anaerobic/anoxic/aerobic-membrane aerated biofilm reactor system. RSC Adv. 10, 28838–28847. doi: 10.1039/d0ra04120c, 35520069 PMC9055795

[ref32] Sun S. P. Nàcher C. P. Merkey B. Nàcher C. P. i. Zhou Q. Xia S.-Q. . (2010). Effective biological nitrogen removal treatment processes for domestic wastewaters with low C/N ratios: a review. Environ. Eng. Sci. 27, 111–126. doi: 10.1089/ees.2009.0100

[ref33] Tang C. C. Zuo W. Tian Y. Sun N. Wang Z. W. Zhang J. (2016). Effect of aeration rate on performance and stability of algal-bacterial symbiosis system to treat domestic wastewater in sequencing batch reactors. Bioresour. Technol. 222, 156–164. doi: 10.1016/j.biortech.2016.09.123, 27718398

[ref34] Wan Y. Huang Z. Zhou L. Li T. Liao C. Yan X. . (2019). Bioelectrochemical ammoniation coupled with microbial electrolysis for nitrogen recovery from nitrate in wastewater. Environ. Sci. Technol. 54, 3002–3011. doi: 10.1021/acs.est.9b05290 31891257

[ref35] Wang J. Tian Q. Cui L. Cheng J. Zhou H. Zhang Y. . (2022). Synergism and mutualistic interactions between microalgae and fungi in fungi-microalgae symbiotic system. Bioresour. Technol. 361:127728. doi: 10.1016/j.biortech.2022.127728, 35932943

[ref36] Wang S. Zhi L. Shan W. Lu H. Xu Q. Li J. (2020). Correlation of extracellular polymeric substances and microbial community structure in denitrification biofilm exposed to adverse conditions. Microb. Biotechnol. 13, 1889–1903. doi: 10.1111/1751-7915.13633, 32700468 PMC7533329

[ref37] Wei J. Huang X. Wang H. Wang F. Liu X. Yan Y. . (2023). Insight into biofilm formation of wastewater treatment processes: nitrogen removal performance and biological mechanism. Sci. Total Environ. 903:166550. doi: 10.1016/j.scitotenv.2023.166550, 37633400

[ref38] Xia Z. Ng H. Y. Xu D. Bae S. (2024). Lumen air pressure regulated multifunctional microbiotas in membrane-aerated biofilm reactors for simultaneous nitrogen removal and antibiotic elimination from aquaculture wastewater. Water Res. 251:121102. doi: 10.1016/j.watres.2024.121102, 38198973

